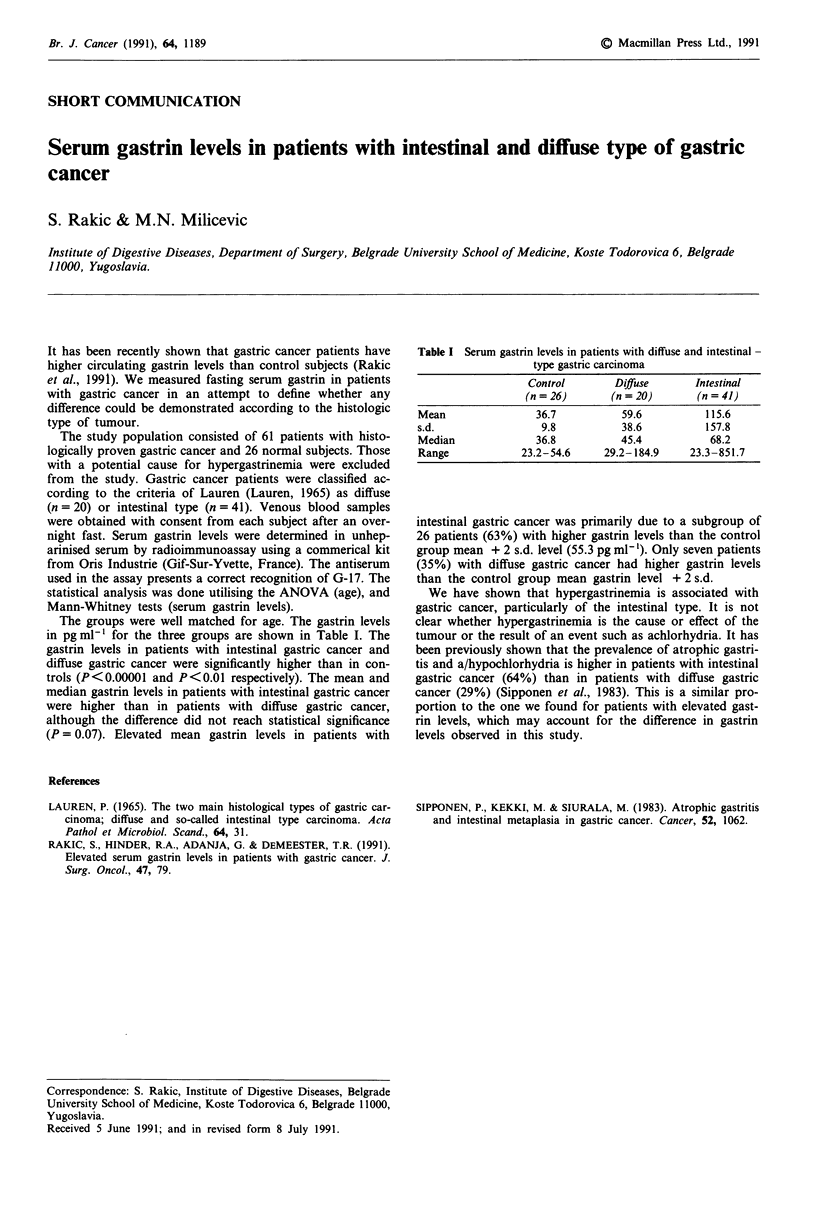# Serum gastrin levels in patients with intestinal and diffuse type of gastric cancer.

**DOI:** 10.1038/bjc.1991.489

**Published:** 1991-12

**Authors:** S. Rakic, M. N. Milicevic

**Affiliations:** Department of Surgery, Belgrade University School of Medicine, Yugoslavia.


 © Macmillan Press Ltd., 1991

SHORT COMMUNICATION

### Serum gastrin levels in patients with intestinal and diffuse type of gastric cancer

S. Rakic & M.N. Milicevic

*Institute of Digestive Diseases, Department of Surgery, Belgrade University School of Medicine, Koste Todorovica 6, Belgrade 11000, Yugoslavia.*

It has been recently shown that gastric cancer patients have higher circulating gastrin levels than control subjects (Rakic *et al.*, 1991). We measured fasting serum gastrin in patients with gastric cancer in an attempt to define whether any difference could be demonstrated according to the histologic type of tumour.

The study population consisted of 61 patients with histologically proven gastric cancer and 26 normal subjects. Those with a potential cause for hypergastrinemia were excluded from the study. Gastric cancer patients were classified according to the criteria of Lauren (Lauren, 1965) as diffuse ($n = 20$) or intestinal type ($n = 41$). Venous blood samples were obtained with consent from each subject after an overnight fast. Serum gastrin levels were determined in unheparinised serum by radioimmunoassay using a commerical kit from Oris Industrie (Gif-Sur-Yvette, France). The antiserum used in the assay presents a correct recognition of G-17. The statistical analysis was done utilising the ANOVA (age), and Mann-Whitney tests (serum gastrin levels).

The groups were well matched for age. The gastrin levels in pg ml$^{-1}$ for the three groups are shown in Table I. The gastrin levels in patients with intestinal gastric cancer and diffuse gastric cancer were significantly higher than in controls ($P < 0.00001$ and $P < 0.01$ respectively). The mean and median gastrin levels in patients with intestinal gastric cancer were higher than in patients with diffuse gastric cancer, although the difference did not reach statistical significance ($P = 0.07$). Elevated mean gastrin levels in patients with intestinal gastric cancer was primarily due to a subgroup of 26 patients (63%) with higher gastrin levels than the control group mean + 2 s.d. level (55.3 pg ml$^{-1}$). Only seven patients (35%) with diffuse gastric cancer had higher gastrin levels than the control group mean gastrin level + 2 s.d.

**Table I** Serum gastrin levels in patients with diffuse and intestinal – type gastric carcinoma

| | *Control* (*n* = 26) | *Diffuse* (*n* = 20) | *Intestinal* (*n* = 41) |
|---|---|---|---|
| Mean | 36.7 | 59.6 | 115.6 |
| s.d. | 9.8 | 38.6 | 157.8 |
| Median | 36.8 | 45.4 | 68.2 |
| Range | 23.2–54.6 | 29.2–184.9 | 23.3–851.7 |

We have shown that hypergastrinemia is associated with gastric cancer, particularly of the intestinal type. It is not clear whether hypergastrinemia is the cause or effect of the tumour or the result of an event such as achlorhydria. It has been previously shown that the prevalence of atrophic gastritis and a/hypochlorhydria is higher in patients with intestinal gastric cancer (64%) than in patients with diffuse gastric cancer (29%) (Sipponen *et al.*, 1983). This is a similar proportion to the one we found for patients with elevated gastrin levels, which may account for the difference in gastrin levels observed in this study.

### References

LAUREN, P. (1965). The two main histological types of gastric carcinoma; diffuse and so-called intestinal type carcinoma. *Acta Pathol et Microbiol. Scand.*, **64**, 31.

RAKIC, S., HINDER, R.A., ADANJA, G. & DEMEESTER, T.R. (1991). Elevated serum gastrin levels in patients with gastric cancer. *J. Surg. Oncol.*, **47**, 79.

SIPPONEN, P., KEKKI, M. & SIURALA, M. (1983). Atrophic gastritis and intestinal metaplasia in gastric cancer. *Cancer*, **52**, 1062.

Correspondence: S. Rakic, Institute of Digestive Diseases, Belgrade University School of Medicine, Koste Todorovica 6, Belgrade 11000, Yugoslavia.
Received 5 June 1991; and in revised form 8 July 1991.